# RETRACTED: Fajdek-Bieda, A.; Wróblewska, A. The Use of Natural Minerals as Reinforcements in Mineral-Reinforced Polymers: A Review of Current Developments and Prospects. *Polymers* 2024, *16*, 2505

**DOI:** 10.3390/polym18222808

**Published:** 2026-11-10

**Authors:** Anna Fajdek-Bieda, Agnieszka Wróblewska

**Affiliations:** 1 Technical Department, Jakub’s from Paradyż Academy in Gorzów Wielkopolski, Chopina 52, 66-400 Gorzów Wielkopolski, Poland; 2 Department of Catalytic and Sorbent Materials Engineering, Faculty of Chemical Technology and Engineering, West Pomeranian University of Technology in Szczecin, Piastów Ave. 42, 71-065 Szczecin, Poland

The journal retracts the review article “The Use of Natural Minerals as Reinforcements in Mineral-Reinforced Polymers: A Review of Current Developments and Prospects” [1] cited above.

Following publication, concerns were brought to the attention of the Editorial Office regarding the accuracy of a reference presented in the publication.

In accordance with the journal’s standard procedures, the Editorial Office and Editorial Board conducted an investigation, which identified and confirmed a significant number of irrelevant citations and instances of content redundancy within this review article. While the authors cooperated with the investigation and provided explanations regarding the identified issues, these explanations were deemed unsatisfactory by the Editorial Board. As a consequence, the Editorial Board lost confidence in the reliability of the review article and has decided to retract this publication in accordance with MDPI’s retraction policy.

This retraction was approved by the Editor-in-Chief of the journal *Polymers*.

The authors have been informed of this retraction and have indicated their disagreement with this decision.
